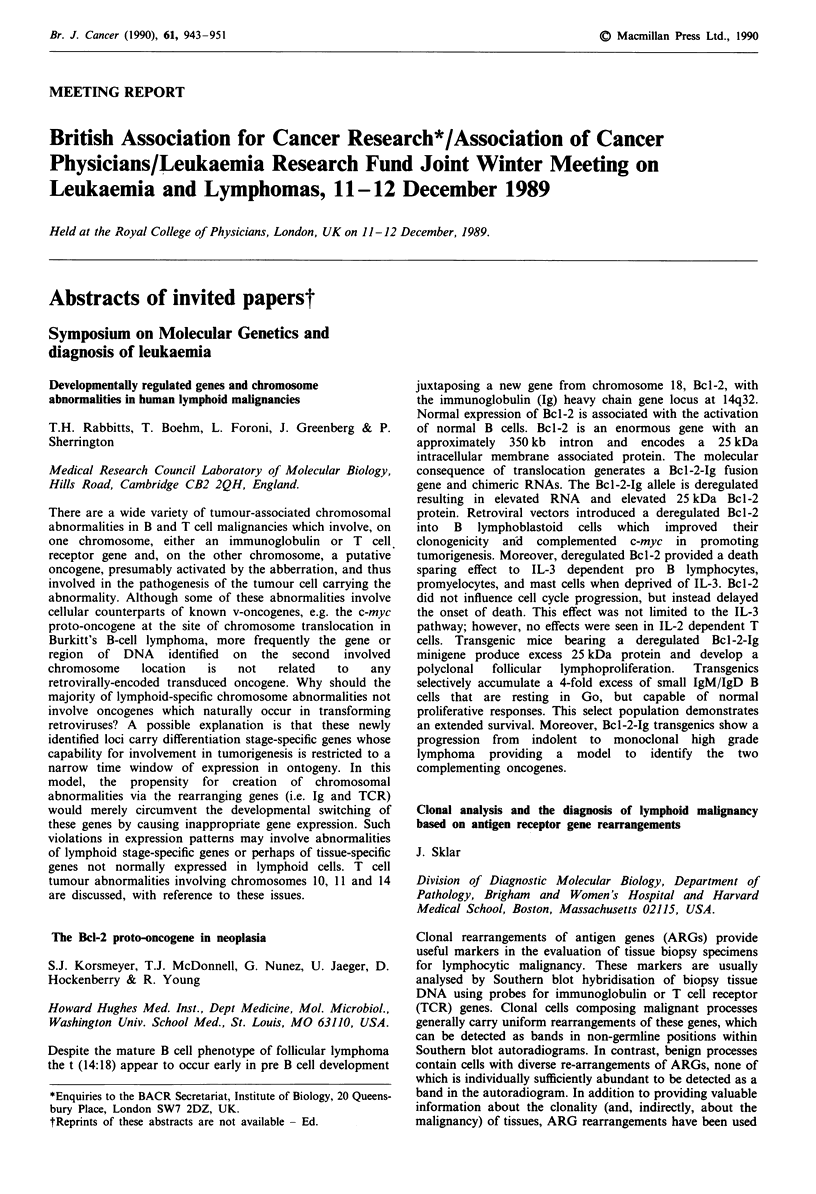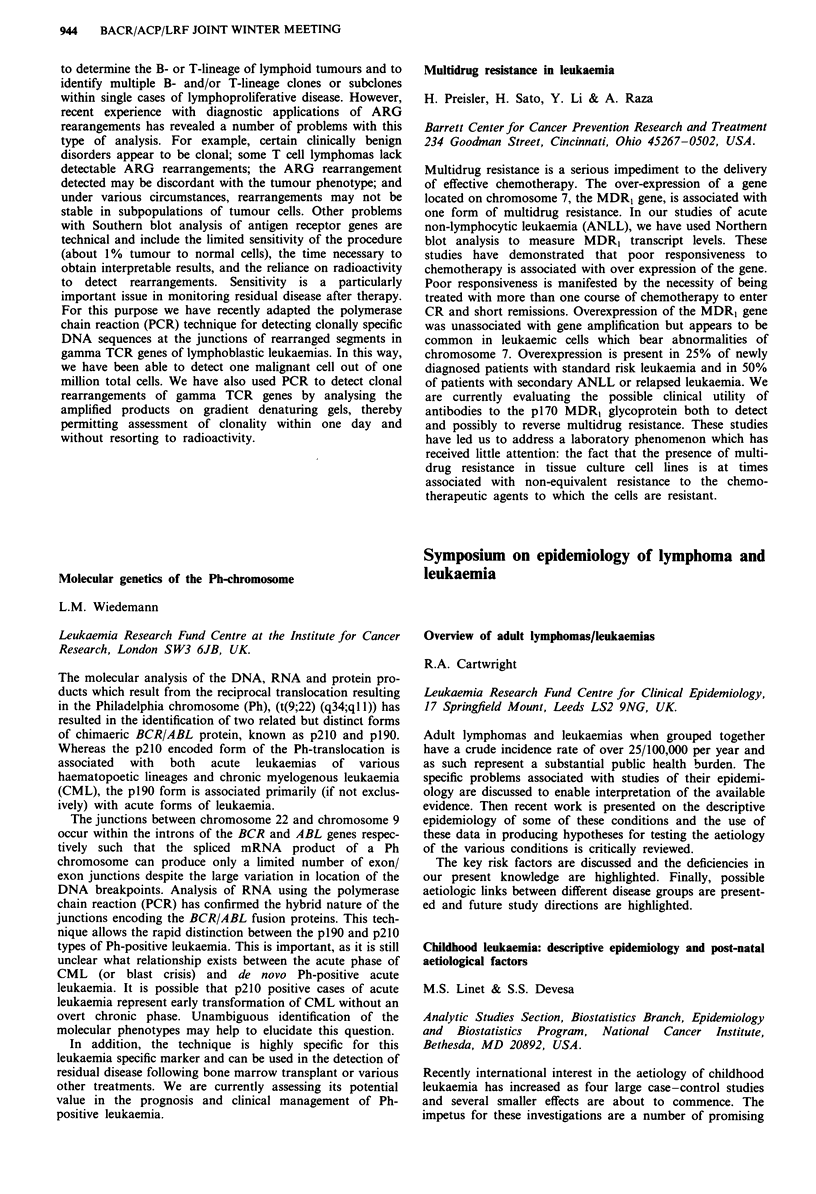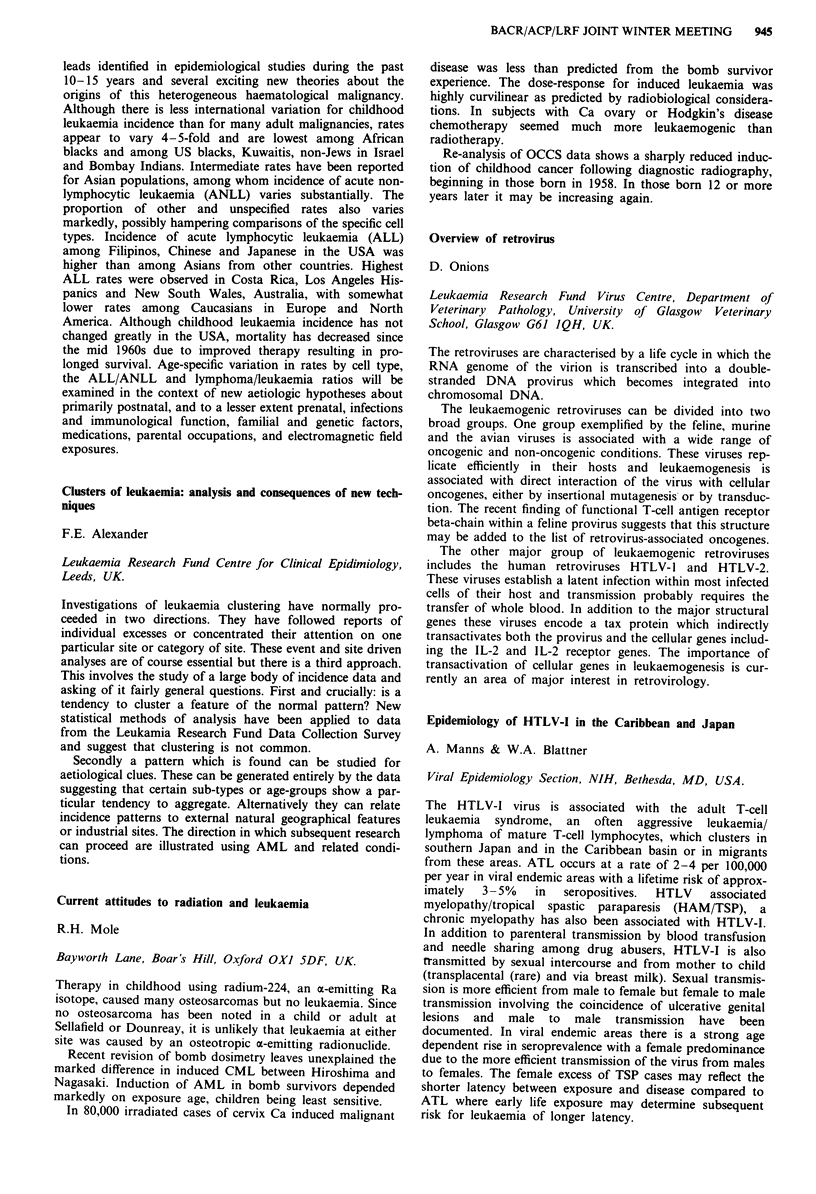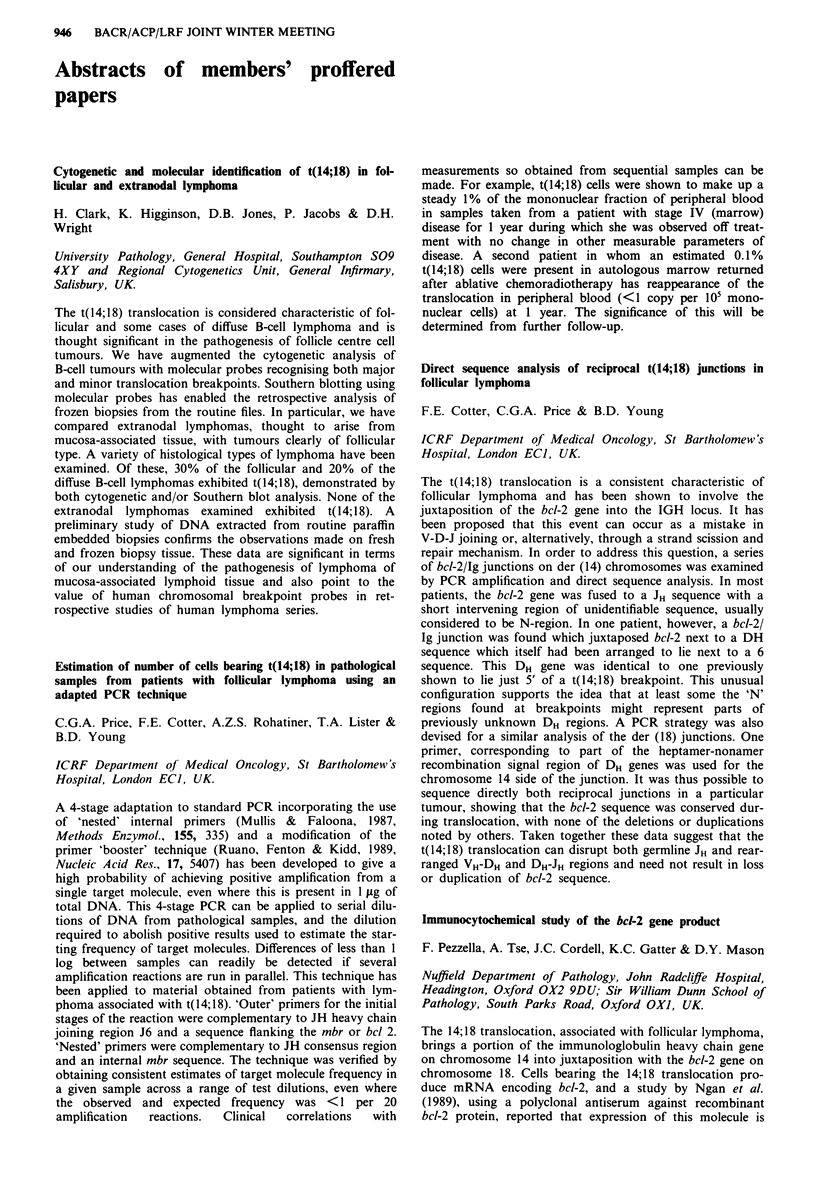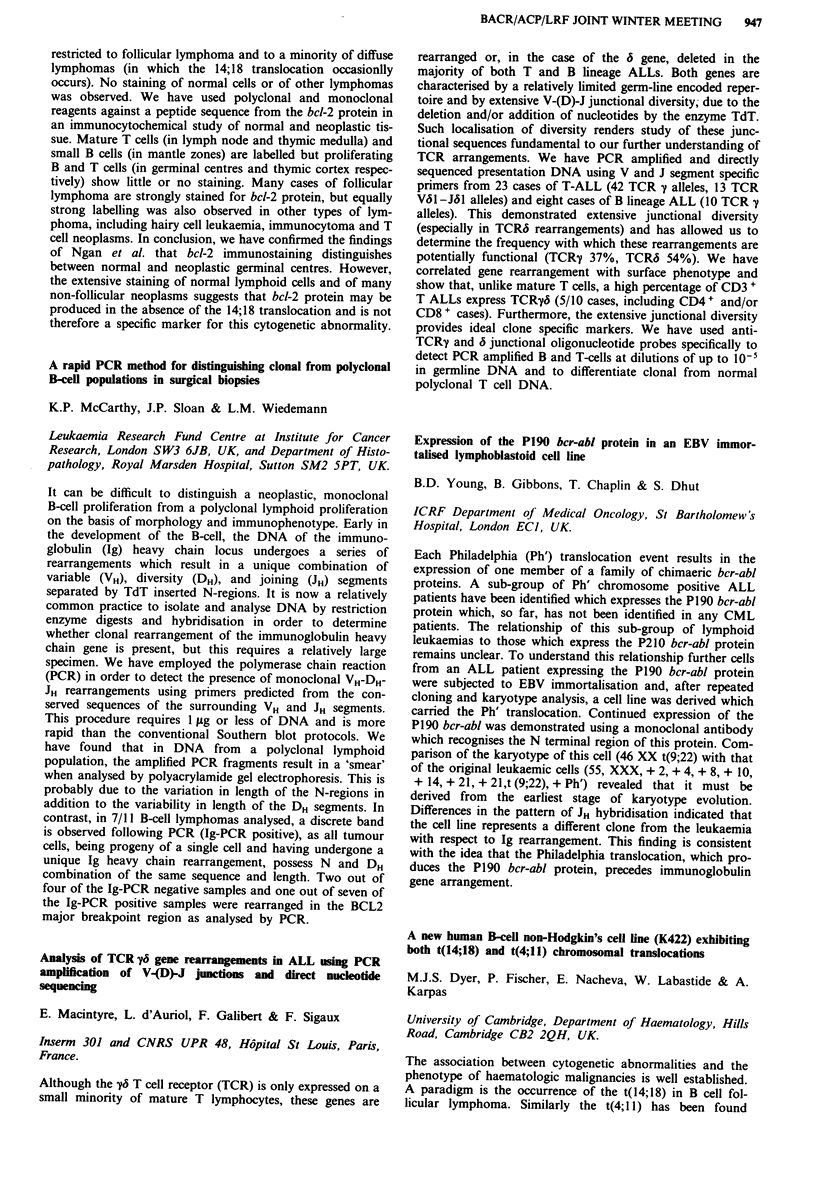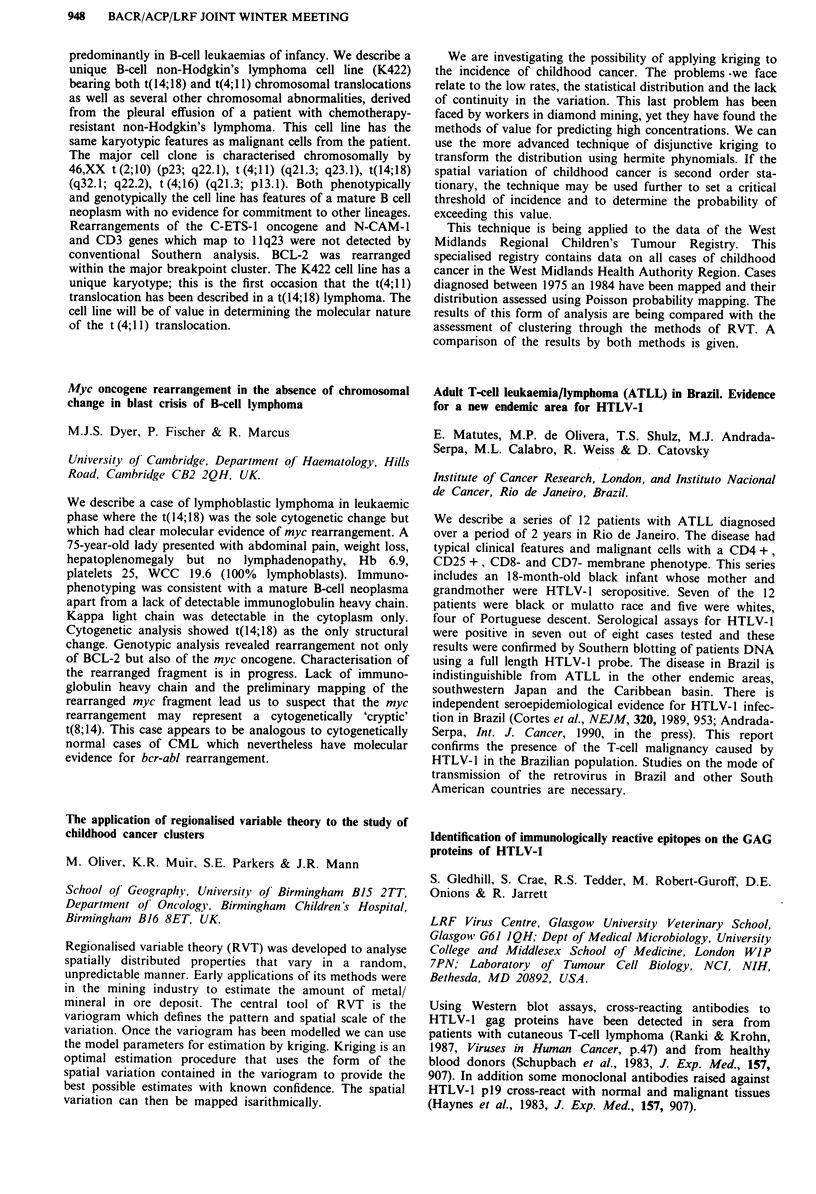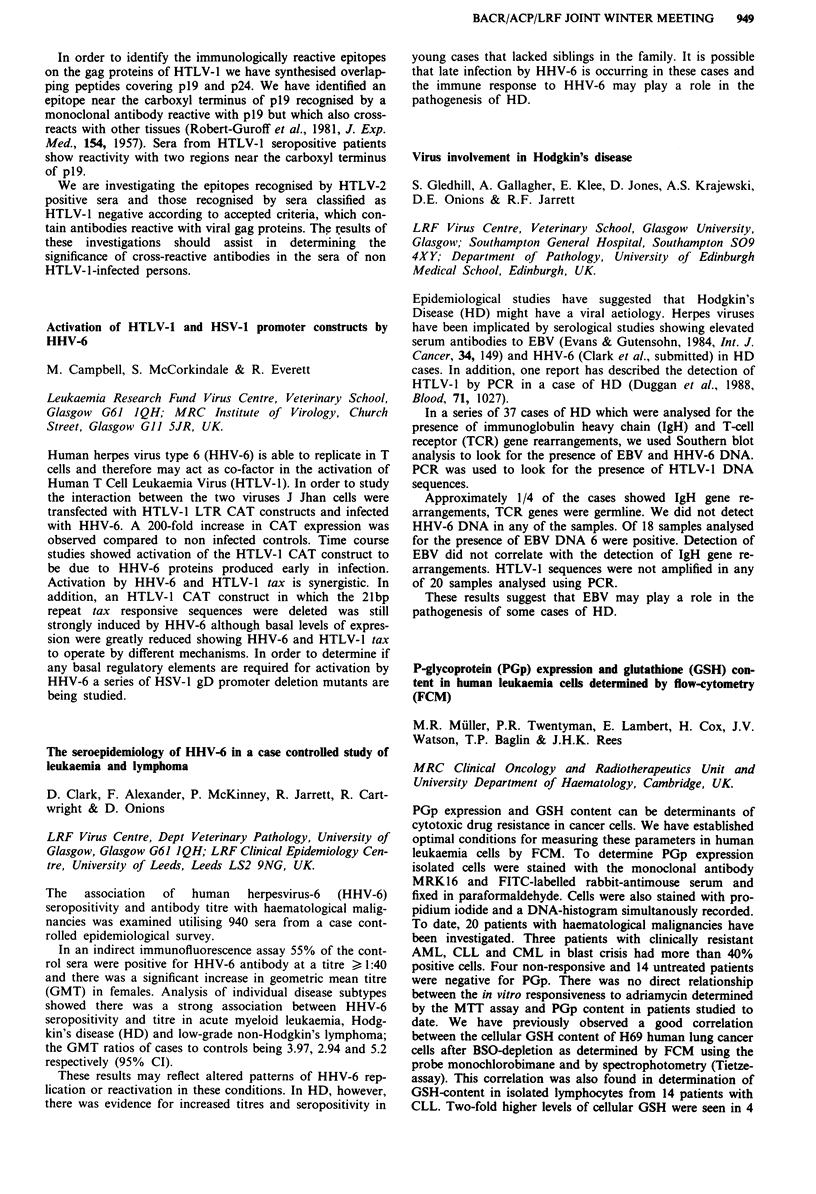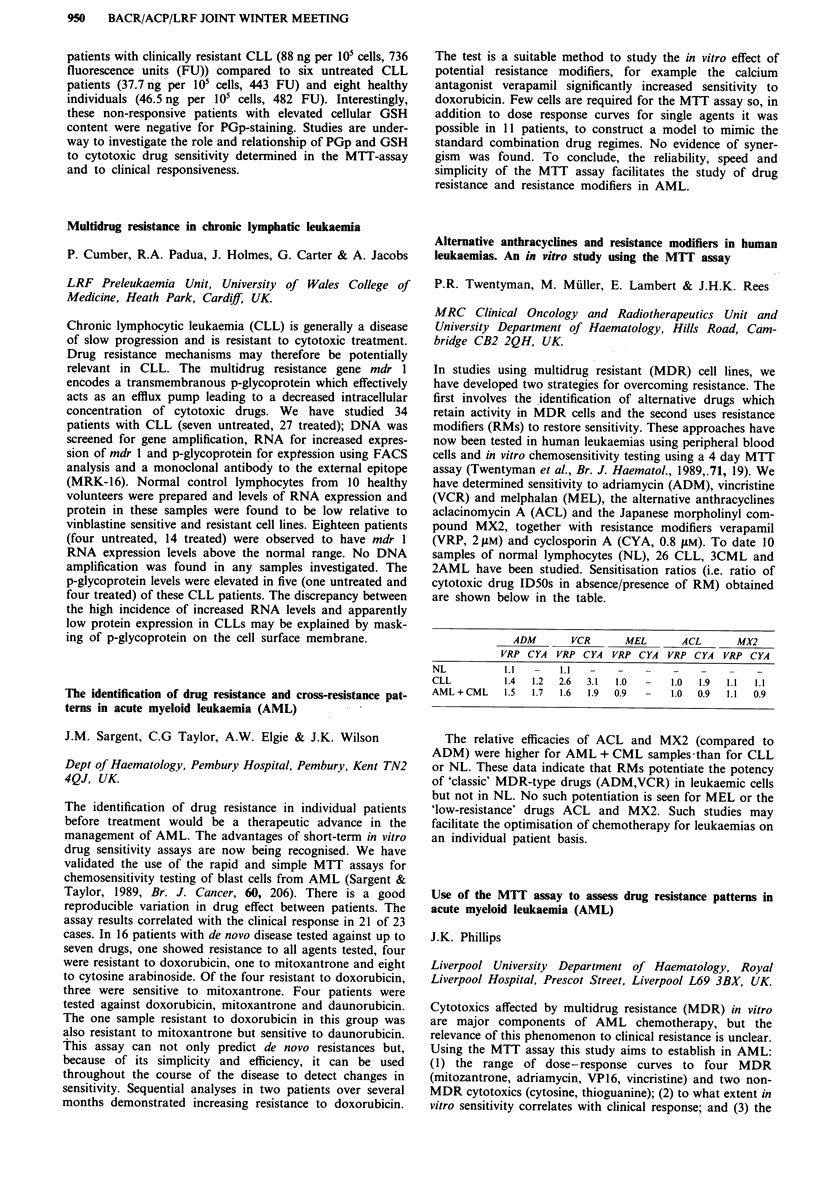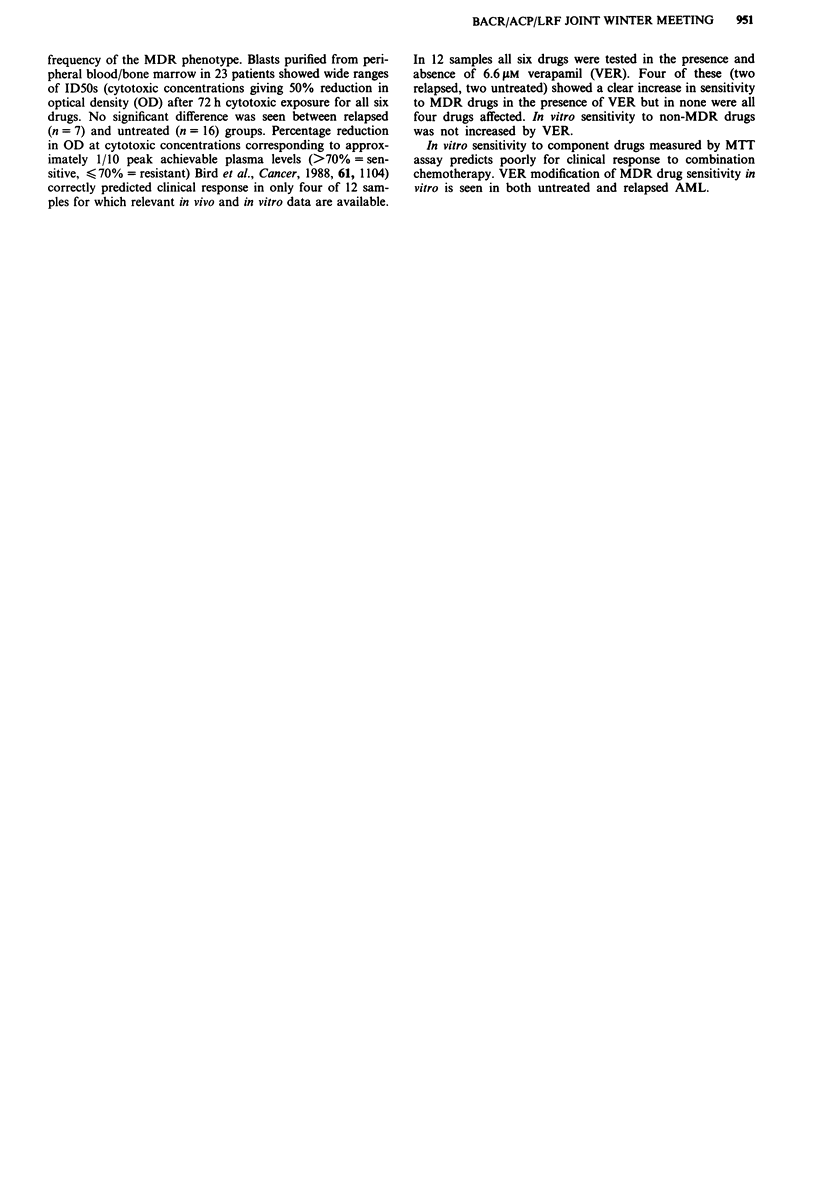# British Association for Cancer Research/ Association of Cancer Physicians/ Leukaemia Research Fund Joint Winter Meeting on Leukaemia and Lymphomas. 11-12 December 1989. Abstracts.

**Published:** 1990-06

**Authors:** 


Br. J. Cancer (1990), 61, 943-951 © Macmillan Press Ltd., 1990

MEETING REPORT

### British Association for Cancer Research*/Association of Cancer Physicians/Leukaemia Research Fund Joint Winter Meeting on Leukaemia and Lymphomas, 11-12 December 1989

*Held at the Royal College of Physicians, London, UK on 11-12 December, 1989.*

### Abstracts of invited papers†

### Symposium on Molecular Genetics and diagnosis of leukaemia

#### Developmentally regulated genes and chromosome abnormalities in human lymphoid malignancies

T.H. Rabbitts, T. Boehm, L. Foroni, J. Greenberg & P. Sherrington

*Medical Research Council Laboratory of Molecular Biology, Hills Road, Cambridge CB2 2QH, England.*

There are a wide variety of tumour-associated chromosomal abnormalities in B and T cell malignancies which involve, on one chromosome, either an immunoglobulin or T cell receptor gene and, on the other chromosome, a putative oncogene, presumably activated by the abberration, and thus involved in the pathogenesis of the tumour cell carrying the abnormality. Although some of these abnormalities involve cellular counterparts of known v-oncogenes, e.g. the c-*myc* proto-oncogene at the site of chromosome translocation in Burkitt's B-cell lymphoma, more frequently the gene or region of DNA identified on the second involved chromosome location is not related to any retrovirally-encoded transduced oncogene. Why should the majority of lymphoid-specific chromosome abnormalities not involve oncogenes which naturally occur in transforming retroviruses? A possible explanation is that these newly identified loci carry differentiation stage-specific genes whose capability for involvement in tumorigenesis is restricted to a narrow time window of expression in ontogeny. In this model, the propensity for creation of chromosomal abnormalities via the rearranging genes (i.e. Ig and TCR) would merely circumvent the developmental switching of these genes by causing inappropriate gene expression. Such violations in expression patterns may involve abnormalities of lymphoid stage-specific genes or perhaps of tissue-specific genes not normally expressed in lymphoid cells. T cell tumour abnormalities involving chromosomes 10, 11 and 14 are discussed, with reference to these issues.

#### The Bcl-2 proto-oncogene in neoplasia

S.J. Korsmeyer, T.J. McDonnell, G. Nunez, U. Jaeger, D. Hockenberry & R. Young

*Howard Hughes Med. Inst., Dept Medicine, Mol. Microbiol., Washington Univ. School Med., St. Louis, MO 63110, USA.*

Despite the mature B cell phenotype of follicular lymphoma the t (14:18) appear to occur early in pre B cell development juxtaposing a new gene from chromosome 18, Bcl-2, with the immunoglobulin (Ig) heavy chain gene locus at 14q32. Normal expression of Bcl-2 is associated with the activation of normal B cells. Bcl-2 is an enormous gene with an approximately 350 kb intron and encodes a 25 kDa intracellular membrane associated protein. The molecular consequence of translocation generates a Bcl-2-Ig fusion gene and chimeric RNAs. The Bcl-2-Ig allele is deregulated resulting in elevated RNA and elevated 25 kDa Bcl-2 protein. Retroviral vectors introduced a deregulated Bcl-2 into B lymphoblastoid cells which improved their clonogenicity and complemented c-*myc* in promoting tumorigenesis. Moreover, deregulated Bcl-2 provided a death sparing effect to IL-3 dependent pro B lymphocytes, promyelocytes, and mast cells when deprived of IL-3. Bcl-2 did not influence cell cycle progression, but instead delayed the onset of death. This effect was not limited to the IL-3 pathway; however, no effects were seen in IL-2 dependent T cells. Transgenic mice bearing a deregulated Bcl-2-Ig minigene produce excess 25 kDa protein and develop a polyclonal follicular lymphoproliferation. Transgenics selectively accumulate a 4-fold excess of small IgM/IgD B cells that are resting in Go, but capable of normal proliferative responses. This select population demonstrates an extended survival. Moreover, Bcl-2-Ig transgenics show a progression from indolent to monoclonal high grade lymphoma providing a model to identify the two complementing oncogenes.

#### Clonal analysis and the diagnosis of lymphoid malignancy based on antigen receptor gene rearrangements

J. Sklar

*Division of Diagnostic Molecular Biology, Department of Pathology, Brigham and Women's Hospital and Harvard Medical School, Boston, Massachusetts 02115, USA.*

Clonal rearrangements of antigen genes (ARGs) provide useful markers in the evaluation of tissue biopsy specimens for lymphocytic malignancy. These markers are usually analysed by Southern blot hybridisation of biopsy tissue DNA using probes for immunoglobulin or T cell receptor (TCR) genes. Clonal cells composing malignant processes generally carry uniform rearrangements of these genes, which can be detected as bands in non-germline positions within Southern blot autoradiograms. In contrast, benign processes contain cells with diverse re-arrangements of ARGs, none of which is individually sufficiently abundant to be detected as a band in the autoradiogram. In addition to providing valuable information about the clonality (and, indirectly, about the malignancy) of tissues, ARG rearrangements have been used

*Enquiries to the BACR Secretariat, Institute of Biology, 20 Queensbury Place, London SW7 2DZ, UK.
†Reprints of these abstracts are not available - Ed.

to determine the B- or T-lineage of lymphoid tumours and to identify multiple B- and/or T-lineage clones or subclones within single cases of lymphoproliferative disease. However, recent experience with diagnostic applications of ARG rearangements has revealed a number of problems with this type of analysis. For example, certain clinically benign disorders appear to be clonal; some T cell lymphomas lack detectable ARG rearrangements; the ARG rearrangement detected may be discordant with the tumour phenotype; and under various circumstances, rearrangements may not be stable in subpopulations of tumour cells. Other problems with Southern blot analysis of antigen receptor genes are technical and include the limited sensitivity of the procedure (about 1% tumour to normal cells), the time necessary to obtain interpretable results, and the reliance on radioactivity to detect rearrangements. Sensitivity is a particularly important issue in monitoring residual disease after therapy. For this purpose we have recently adapted the polymerase chain reaction (PCR) technique for detecting clonally specific DNA sequences at the junctions of rearranged segments in gamma TCR genes of lymphoblastic leukaemias. In this way, we have been able to detect one malignant cell out of one million total cells. We have also used PCR to detect clonal rearrangements of gamma TCR genes by analysing the amplified products on gradient denaturing gels, thereby permitting assessment of clonality within one day and without resorting to radioactivity.

### Molecular genetics of the Ph-chromosome

L.M. Wiedemann

*Leukaemia Research Fund Centre at the Institute for Cancer Research, London SW3 6JB, UK.*

The molecular analysis of the DNA, RNA and protein products which result from the reciprocal translocation resulting in the Philadelphia chromosome (Ph), (t(9;22) (q34;q11)) has resulted in the identification of two related but distinct forms of chimaeric *BCR/ABL* protein, known as p210 and p190. Whereas the p210 encoded form of the Ph-translocation is associated with both acute leukaemias of various haematopoetic lineages and chronic myelogenous leukaemia (CML), the p190 form is associated primarily (if not exclusively) with acute forms of leukaemia.

The junctions between chromosome 22 and chromosome 9 occur within the introns of the *BCR* and *ABL* genes respectively such that the spliced mRNA product of a Ph chromosome can produce only a limited number of exon/exon junctions despite the large variation in location of the DNA breakpoints. Analysis of RNA using the polymerase chain reaction (PCR) has confirmed the hybrid nature of the junctions encoding the *BCR/ABL* fusion proteins. This technique allows the rapid distinction between the p190 and p210 types of Ph-positive leukaemia. This is important, as it is still unclear what relationship exists between the acute phase of CML (or blast crisis) and *de novo* Ph-positive acute leukaemia. It is possible that p210 positive cases of acute leukaemia represent early transformation of CML without an overt chronic phase. Unambiguous identification of the molecular phenotypes may help to elucidate this question.

In addition, the technique is highly specific for this leukaemia specific marker and can be used in the detection of residual disease following bone marrow transplant or various other treatments. We are currently assessing its potential value in the prognosis and clinical management of Ph-positive leukaemia.

### Multidrug resistance in leukaemia

H. Preisler, H. Sato, Y. Li & A. Raza

*Barrett Center for Cancer Prevention Research and Treatment 234 Goodman Street, Cincinnati, Ohio 45267-0502, USA.*

Multidrug resistance is a serious impediment to the delivery of effective chemotherapy. The over-expression of a gene located on chromosome 7, the $MDR_1$ gene, is associated with one form of multidrug resistance. In our studies of acute non-lymphocytic leukaemia (ANLL), we have used Northern blot analysis to measure $MDR_1$ transcript levels. These studies have demonstrated that poor responsiveness to chemotherapy is associated with over expression of the gene. Poor responsiveness is manifested by the necessity of being treated with more than one course of chemotherapy to enter CR and short remissions. Overexpression of the $MDR_1$ gene was unassociated with gene amplification but appears to be common in leukaemic cells which bear abnormalities of chromosome 7. Overexpression is present in 25% of newly diagnosed patients with standard risk leukaemia and in 50% of patients with secondary ANLL or relapsed leukaemia. We are currently evaluating the possible clinical utility of antibodies to the p170 $MDR_1$ glycoprotein both to detect and possibly to reverse multidrug resistance. These studies have led us to address a laboratory phenomenon which has received little attention: the fact that the presence of multidrug resistance in tissue culture cell lines is at times associated with non-equivalent resistance to the chemotherapeutic agents to which the cells are resistant.

### Symposium on epidemiology of lymphoma and leukaemia

### Overview of adult lymphomas/leukaemias

R.A. Cartwright

*Leukaemia Research Fund Centre for Clinical Epidemiology, 17 Springfield Mount, Leeds LS2 9NG, UK.*

Adult lymphomas and leukaemias when grouped together have a crude incidence rate of over 25/100,000 per year and as such represent a substantial public health burden. The specific problems associated with studies of their epidemiology are discussed to enable interpretation of the available evidence. Then recent work is presented on the descriptive epidemiology of some of these conditions and the use of these data in producing hypotheses for testing the aetiology of the various conditions is critically reviewed.

The key risk factors are discussed and the deficiencies in our present knowledge are highlighted. Finally, possible aetiologic links between different disease groups are presented and future study directions are highlighted.

### Childhood leukaemia: descriptive epidemiology and post-natal aetiological factors

M.S. Linet & S.S. Devesa

*Analytic Studies Section, Biostatistics Branch, Epidemiology and Biostatistics Program, National Cancer Institute, Bethesda, MD 20892, USA.*

Recently international interest in the aetiology of childhood leukaemia has increased as four large case-control studies and several smaller effects are about to commence. The impetus for these investigations are a number of promising leads identified in epidemiological studies during the past 10–15 years and several exciting new theories about the origins of this heterogeneous haematological malignancy. Although there is less international variation for childhood leukaemia incidence than for many adult malignancies, rates appear to vary 4–5-fold and are lowest among African blacks and among US blacks, Kuwaitis, non-Jews in Israel and Bombay Indians. Intermediate rates have been reported for Asian populations, among whom incidence of acute non-lymphocytic leukaemia (ANLL) varies substantially. The proportion of other and unspecified rates also varies markedly, possibly hampering comparisons of the specific cell types. Incidence of acute lymphocytic leukaemia (ALL) among Filipinos, Chinese and Japanese in the USA was higher than among Asians from other countries. Highest ALL rates were observed in Costa Rica, Los Angeles Hispanics and New South Wales, Australia, with somewhat lower rates among Caucasians in Europe and North America. Although childhood leukaemia incidence has not changed greatly in the USA, mortality has decreased since the mid 1960s due to improved therapy resulting in prolonged survival. Age-specific variation in rates by cell type, the ALL/ANLL and lymphoma/leukaemia ratios will be examined in the context of new aetiologic hypotheses about primarily postnatal, and to a lesser extent prenatal, infections and immunological function, familial and genetic factors, medications, parental occupations, and electromagnetic field exposures.

### Clusters of leukaemia: analysis and consequences of new techniques

F.E. Alexander

*Leukaemia Research Fund Centre for Clinical Epidimiology, Leeds, UK.*

Investigations of leukaemia clustering have normally proceeded in two directions. They have followed reports of individual excesses or concentrated their attention on one particular site or category of site. These event and site driven analyses are of course essential but there is a third approach. This involves the study of a large body of incidence data and asking of it fairly general questions. First and crucially: is a tendency to cluster a feature of the normal pattern? New statistical methods of analysis have been applied to data from the Leukamia Research Fund Data Collection Survey and suggest that clustering is not common.

Secondly a pattern which is found can be studied for aetiological clues. These can be generated entirely by the data suggesting that certain sub-types or age-groups show a particular tendency to aggregate. Alternatively they can relate incidence patterns to external natural geographical features or industrial sites. The direction in which subsequent research can proceed are illustrated using AML and related conditions.

### Current attitudes to radiation and leukaemia

R.H. Mole

*Bayworth Lane, Boar's Hill, Oxford OX1 5DF, UK.*

Therapy in childhood using radium-224, an α-emitting Ra isotope, caused many osteosarcomas but no leukaemia. Since no osteosarcoma has been noted in a child or adult at Sellafield or Dounreay, it is unlikely that leukaemia at either site was caused by an osteotropic α-emitting radionuclide.

Recent revision of bomb dosimetry leaves unexplained the marked difference in induced CML between Hiroshima and Nagasaki. Induction of AML in bomb survivors depended markedly on exposure age, children being least sensitive.

In 80,000 irradiated cases of cervix Ca induced malignant disease was less than predicted from the bomb survivor experience. The dose-response for induced leukaemia was highly curvilinear as predicted by radiobiological considerations. In subjects with Ca ovary or Hodgkin's disease chemotherapy seemed much more leukaemogenic than radiotherapy.

Re-analysis of OCCS data shows a sharply reduced induction of childhood cancer following diagnostic radiography, beginning in those born in 1958. In those born 12 or more years later it may be increasing again.

### Overview of retrovirus

D. Onions

*Leukaemia Research Fund Virus Centre, Department of Veterinary Pathology, University of Glasgow Veterinary School, Glasgow G61 1QH, UK.*

The retroviruses are characterised by a life cycle in which the RNA genome of the virion is transcribed into a double-stranded DNA provirus which becomes integrated into chromosomal DNA.

The leukaemogenic retroviruses can be divided into two broad groups. One group exemplified by the feline, murine and the avian viruses is associated with a wide range of oncogenic and non-oncogenic conditions. These viruses replicate efficiently in their hosts and leukaemogenesis is associated with direct interaction of the virus with cellular oncogenes, either by insertional mutagenesis or by transduction. The recent finding of functional T-cell antigen receptor beta-chain within a feline provirus suggests that this structure may be added to the list of retrovirus-associated oncogenes.

The other major group of leukaemogenic retroviruses includes the human retroviruses HTLV-1 and HTLV-2. These viruses establish a latent infection within most infected cells of their host and transmission probably requires the transfer of whole blood. In addition to the major structural genes these viruses encode a tax protein which indirectly transactivates both the provirus and the cellular genes including the IL-2 and IL-2 receptor genes. The importance of transactivation of cellular genes in leukaemogenesis is currently an area of major interest in retrovirology.

### Epidemiology of HTLV-I in the Caribbean and Japan

A. Manns & W.A. Blattner

*Viral Epidemiology Section, NIH, Bethesda, MD, USA.*

The HTLV-I virus is associated with the adult T-cell leukaemia syndrome, an often aggressive leukaemia/lymphoma of mature T-cell lymphocytes, which clusters in southern Japan and in the Caribbean basin or in migrants from these areas. ATL occurs at a rate of 2–4 per 100,000 per year in viral endemic areas with a lifetime risk of approximately 3–5% in seropositives. HTLV associated myelopathy/tropical spastic paraparesis (HAM/TSP), a chronic myelopathy has also been associated with HTLV-I. In addition to parenteral transmission by blood transfusion and needle sharing among drug abusers, HTLV-I is also transmitted by sexual intercourse and from mother to child (transplacental (rare) and via breast milk). Sexual transmission is more efficient from male to female but female to male transmission involving the coincidence of ulcerative genital lesions and male to male transmission have been documented. In viral endemic areas there is a strong age dependent rise in seroprevalence with a female predominance due to the more efficient transmission of the virus from males to females. The female excess of TSP cases may reflect the shorter latency between exposure and disease compared to ATL where early life exposure may determine subsequent risk for leukaemia of longer latency.

### Abstracts of members' proffered papers

### Cytogenetic and molecular identification of t(14;18) in follicular and extranodal lymphoma

H. Clark, K. Higginson, D.B. Jones, P. Jacobs & D.H. Wright

*University Pathology, General Hospital, Southampton SO9 4XY and Regional Cytogenetics Unit, General Infirmary, Salisbury, UK.*

The t(14;18) translocation is considered characteristic of follicular and some cases of diffuse B-cell lymphoma and is thought significant in the pathogenesis of follicle centre cell tumours. We have augmented the cytogenetic analysis of B-cell tumours with molecular probes recognising both major and minor translocation breakpoints. Southern blotting using molecular probes has enabled the retrospective analysis of frozen biopsies from the routine files. In particular, we have compared extranodal lymphomas, thought to arise from mucosa-associated tissue, with tumours clearly of follicular type. A variety of histological types of lymphoma have been examined. Of these, 30% of the follicular and 20% of the diffuse B-cell lymphomas exhibited t(14;18), demonstrated by both cytogenetic and/or Southern blot analysis. None of the extranodal lymphomas examined exhibited t(14;18). A preliminary study of DNA extracted from routine paraffin embedded biopsies confirms the observations made on fresh and frozen biopsy tissue. These data are significant in terms of our understanding of the pathogenesis of lymphoma of mucosa-associated lymphoid tissue and also point to the value of human chromosomal breakpoint probes in retrospective studies of human lymphoma series.

### Estimation of number of cells bearing t(14;18) in pathological samples from patients with follicular lymphoma using an adapted PCR technique

C.G.A. Price, F.E. Cotter, A.Z.S. Rohatiner, T.A. Lister & B.D. Young

*ICRF Department of Medical Oncology, St Bartholomew's Hospital, London EC1, UK.*

A 4-stage adaptation to standard PCR incorporating the use of 'nested' internal primers (Mullis & Faloona, 1987, *Methods Enzymol.*, **155**, 335) and a modification of the primer 'booster' technique (Ruano, Fenton & Kidd, 1989, *Nucleic Acid Res.*, **17**, 5407) has been developed to give a high probability of achieving positive amplification from a single target molecule, even where this is present in 1 μg of total DNA. This 4-stage PCR can be applied to serial dilutions of DNA from pathological samples, and the dilution required to abolish positive results used to estimate the starting frequency of target molecules. Differences of less than 1 log between samples can readily be detected if several amplification reactions are run in parallel. This technique has been applied to material obtained from patients with lymphoma associated with t(14;18). 'Outer' primers for the initial stages of the reaction were complementary to JH heavy chain joining region J6 and a sequence flanking the *mbr* or *bcl* 2. 'Nested' primers were complementary to JH consensus region and an internal *mbr* sequence. The technique was verified by obtaining consistent estimates of target molecule frequency in a given sample across a range of test dilutions, even where the observed and expected frequency was <1 per 20 amplification reactions. Clinical correlations with measurements so obtained from sequential samples can be made. For example, t(14;18) cells were shown to make up a steady 1% of the mononuclear fraction of peripheral blood in samples taken from a patient with stage IV (marrow) disease for 1 year during which she was observed off treatment with no change in other measurable parameters of disease. A second patient in whom an estimated 0.1% t(14;18) cells were present in autologous marrow returned after ablative chemoradiotherapy has reappearance of the translocation in peripheral blood (<1 copy per $10^5$ mononuclear cells) at 1 year. The significance of this will be determined from further follow-up.

### Direct sequence analysis of reciprocal t(14;18) junctions in follicular lymphoma

F.E. Cotter, C.G.A. Price & B.D. Young

*ICRF Department of Medical Oncology, St Bartholomew's Hospital, London EC1, UK.*

The t(14;18) translocation is a consistent characteristic of follicular lymphoma and has been shown to involve the juxtaposition of the *bcl*-2 gene into the IGH locus. It has been proposed that this event can occur as a mistake in V-D-J joining or, alternatively, through a strand scission and repair mechanism. In order to address this question, a series of *bcl*-2/Ig junctions on der (14) chromosomes was examined by PCR amplification and direct sequence analysis. In most patients, the *bcl*-2 gene was fused to a $J_H$ sequence with a short intervening region of unidentifiable sequence, usually considered to be N-region. In one patient, however, a *bcl*-2/Ig junction was found which juxtaposed *bcl*-2 next to a DH sequence which itself had been arranged to lie next to a 6 sequence. This $D_H$ gene was identical to one previously shown to lie just 5' of a t(14;18) breakpoint. This unusual configuration supports the idea that at least some the 'N' regions found at breakpoints might represent parts of previously unknown $D_H$ regions. A PCR strategy was also devised for a similar analysis of the der (18) junctions. One primer, corresponding to part of the heptamer-nonamer recombination signal region of $D_H$ genes was used for the chromosome 14 side of the junction. It was thus possible to sequence directly both reciprocal junctions in a particular tumour, showing that the *bcl*-2 sequence was conserved during translocation, with none of the deletions or duplications noted by others. Taken together these data suggest that the t(14;18) translocation can disrupt both germline $J_H$ and rearranged $V_H$-$D_H$ and $D_H$-$J_H$ regions and need not result in loss or duplication of *bcl*-2 sequence.

### Immunocytochemical study of the *bcl*-2 gene product

F. Pezzella, A. Tse, J.C. Cordell, K.C. Gatter & D.Y. Mason

*Nuffield Department of Pathology, John Radcliffe Hospital, Headington, Oxford OX2 9DU; Sir William Dunn School of Pathology, South Parks Road, Oxford OX1, UK.*

The 14;18 translocation, associated with follicular lymphoma, brings a portion of the immunologlobulin heavy chain gene on chromosome 14 into juxtaposition with the *bcl*-2 gene on chromosome 18. Cells bearing the 14;18 translocation produce mRNA encoding *bcl*-2, and a study by Ngan *et al.* (1989), using a polyclonal antiserum against recombinant *bcl*-2 protein, reported that expression of this molecule is restricted to follicular lymphoma and to a minority of diffuse lymphomas (in which the 14;18 translocation occasionlly occurs). No staining of normal cells or of other lymphomas was observed. We have used polyclonal and monoclonal reagents against a peptide sequence from the *bcl*-2 protein in an immunocytochemical study of normal and neoplastic tissue. Mature T cells (in lymph node and thymic medulla) and small B cells (in mantle zones) are labelled but proliferating B and T cells (in germinal centres and thymic cortex respectively) show little or no staining. Many cases of follicular lymphoma are strongly stained for *bcl*-2 protein, but equally strong labelling was also observed in other types of lymphoma, including hairy cell leukaemia, immunocytoma and T cell neoplasms. In conclusion, we have confirmed the findings of Ngan *et al.* that *bcl*-2 immunostaining distinguishes between normal and neoplastic germinal centres. However, the extensive staining of normal lymphoid cells and of many non-follicular neoplasms suggests that *bcl*-2 protein may be produced in the absence of the 14;18 translocation and is not therefore a specific marker for this cytogenetic abnormality.

**A rapid PCR method for distinguishing clonal from polyclonal B-cell populations in surgical biopsies**

K.P. McCarthy, J.P. Sloan & L.M. Wiedemann

*Leukaemia Research Fund Centre at Institute for Cancer Research, London SW3 6JB, UK, and Department of Histopathology, Royal Marsden Hospital, Sutton SM2 5PT, UK.*

It can be difficult to distinguish a neoplastic, monoclonal B-cell proliferation from a polyclonal lymphoid proliferation on the basis of morphology and immunophenotype. Early in the development of the B-cell, the DNA of the immunoglobulin (Ig) heavy chain locus undergoes a series of rearrangements which result in a unique combination of variable ($V_H$), diversity ($D_H$), and joining ($J_H$) segments separated by TdT inserted N-regions. It is now a relatively common practice to isolate and analyse DNA by restriction enzyme digests and hybridisation in order to determine whether clonal rearrangement of the immunoglobulin heavy chain gene is present, but this requires a relatively large specimen. We have employed the polymerase chain reaction (PCR) in order to detect the presence of monoclonal $V_H$-$D_H$-$J_H$ rearrangements using primers predicted from the conserved sequences of the surrounding $V_H$ and $J_H$ segments. This procedure requires 1 μg or less of DNA and is more rapid than the conventional Southern blot protocols. We have found that in DNA from a polyclonal lymphoid population, the amplified PCR fragments result in a 'smear' when analysed by polyacrylamide gel electrophoresis. This is probably due to the variation in length of the N-regions in addition to the variability in length of the $D_H$ segments. In contrast, in 7/11 B-cell lymphomas analysed, a discrete band is observed following PCR (Ig-PCR positive), as all tumour cells, being progeny of a single cell and having undergone a unique Ig heavy chain rearrangement, possess N and $D_H$ combination of the same sequence and length. Two out of four of the Ig-PCR negative samples and one out of seven of the Ig-PCR positive samples were rearranged in the BCL2 major breakpoint region as analysed by PCR.

**Analysis of TCR γδ gene rearrangements in ALL using PCR amplification of V-(D)-J junctions and direct nucleotide sequencing**

E. Macintyre, L. d'Auriol, F. Galibert & F. Sigaux

*Inserm 301 and CNRS UPR 48, Hôpital St Louis, Paris, France.*

Although the γδ T cell receptor (TCR) is only expressed on a small minority of mature T lymphocytes, these genes are rearranged or, in the case of the δ gene, deleted in the majority of both T and B lineage ALLs. Both genes are characterised by a relatively limited germ-line encoded repertoire and by extensive V-(D)-J junctional diversity, due to the deletion and/or addition of nucleotides by the enzyme TdT. Such localisation of diversity renders study of these junctional sequences fundamental to our further understanding of TCR arrangements. We have PCR amplified and directly sequenced presentation DNA using V and J segment specific primers from 23 cases of T-ALL (42 TCR γ alleles, 13 TCR Vδ1-Jδ1 alleles) and eight cases of B lineage ALL (10 TCR γ alleles). This demonstrated extensive junctional diversity (especially in TCRδ rearrangements) and has allowed us to determine the frequency with which these rearrangements are potentially functional (TCRγ 37%, TCRδ 54%). We have correlated gene rearrangement with surface phenotype and show that, unlike mature T cells, a high percentage of $CD3^+$ T ALLs express TCRγδ (5/10 cases, including $CD4^+$ and/or $CD8^+$ cases). Furthermore, the extensive junctional diversity provides ideal clone specific markers. We have used anti-TCRγ and δ junctional oligonucleotide probes specifically to detect PCR amplified B and T-cells at dilutions of up to $10^{-5}$ in germline DNA and to differentiate clonal from normal polyclonal T cell DNA.

**Expression of the P190 *bcr-abl* protein in an EBV immortalised lymphoblastoid cell line**

B.D. Young, B. Gibbons, T. Chaplin & S. Dhut

*ICRF Department of Medical Oncology, St Bartholomew's Hospital, London EC1, UK.*

Each Philadelphia (Ph') translocation event results in the expression of one member of a family of chimaeric *bcr-abl* proteins. A sub-group of Ph' chromosome positive ALL patients have been identified which expresses the P190 *bcr-abl* protein which, so far, has not been identified in any CML patients. The relationship of this sub-group of lymphoid leukaemias to those which express the P210 *bcr-abl* protein remains unclear. To understand this relationship further cells from an ALL patient expressing the P190 *bcr-abl* protein were subjected to EBV immortalisation and, after repeated cloning and karyotype analysis, a cell line was derived which carried the Ph' translocation. Continued expression of the P190 *bcr-abl* was demonstrated using a monoclonal antibody which recognises the N terminal region of this protein. Comparison of the karyotype of this cell (46 XX t(9;22) with that of the original leukaemic cells (55, XXX, + 2, + 4, + 8, + 10, + 14, + 21, + 21,t (9;22), + Ph') revealed that it must be derived from the earliest stage of karyotype evolution. Differences in the pattern of $J_H$ hybridisation indicated that the cell line represents a different clone from the leukaemia with respect to Ig rearrangement. This finding is consistent with the idea that the Philadelphia translocation, which produces the P190 *bcr-abl* protein, precedes immunoglobulin gene arrangement.

**A new human B-cell non-Hodgkin's cell line (K422) exhibiting both t(14;18) and t(4;11) chromosomal translocations**

M.J.S. Dyer, P. Fischer, E. Nacheva, W. Labastide & A. Karpas

*University of Cambridge, Department of Haematology, Hills Road, Cambridge CB2 2QH, UK.*

The association between cytogenetic abnormalities and the phenotype of haematologic malignancies is well established. A paradigm is the occurrence of the t(14;18) in B cell follicular lymphoma. Similarly the t(4;11) has been found predominantly in B-cell leukaemias of infancy. We describe a unique B-cell non-Hodgkin's lymphoma cell line (K422) bearing both t(14;18) and t(4;11) chromosomal translocations as well as several other chromosomal abnormalities, derived from the pleural effusion of a patient with chemotherapy-resistant non-Hodgkin's lymphoma. This cell line has the same karyotypic features as malignant cells from the patient. The major cell clone is characterised chromosomally by 46,XX t (2;10) (p23; q22.1), t (4;11) (q21.3; q23.1), t(14;18) (q32.1; q22.2), t (4;16) (q21.3; p13.1). Both phenotypically and genotypically the cell line has features of a mature B cell neoplasm with no evidence for commitment to other lineages. Rearrangements of the C-ETS-1 oncogene and N-CAM-1 and CD3 genes which map to 11q23 were not detected by conventional Southern analysis. BCL-2 was rearranged within the major breakpoint cluster. The K422 cell line has a unique karyotype; this is the first occasion that the t(4;11) translocation has been described in a t(14;18) lymphoma. The cell line will be of value in determining the molecular nature of the t (4;11) translocation.

### *Myc* oncogene rearrangement in the absence of chromosomal change in blast crisis of B-cell lymphoma

M.J.S. Dyer, P. Fischer & R. Marcus

*University of Cambridge, Department of Haematology, Hills Road, Cambridge CB2 2QH, UK.*

We describe a case of lymphoblastic lymphoma in leukaemic phase where the t(14;18) was the sole cytogenetic change but which had clear molecular evidence of *myc* rearrangement. A 75-year-old lady presented with abdominal pain, weight loss, hepatoplenomegaly but no lymphadenopathy, Hb 6.9, platelets 25, WCC 19.6 (100% lymphoblasts). Immunophenotyping was consistent with a mature B-cell neoplasma apart from a lack of detectable immunoglobulin heavy chain. Kappa light chain was detectable in the cytoplasm only. Cytogenetic analysis showed t(14;18) as the only structural change. Genotypic analysis revealed rearrangement not only of BCL-2 but also of the *myc* oncogene. Characterisation of the rearranged fragment is in progress. Lack of immunoglobulin heavy chain and the preliminary mapping of the rearranged *myc* fragment lead us to suspect that the *myc* rearrangement may represent a cytogenetically 'cryptic' t(8;14). This case appears to be analogous to cytogenetically normal cases of CML which nevertheless have molecular evidence for *bcr-abl* rearrangement.

### The application of regionalised variable theory to the study of childhood cancer clusters

M. Oliver, K.R. Muir, S.E. Parkers & J.R. Mann

*School of Geography, University of Birmingham B15 2TT, Department of Oncology, Birmingham Children's Hospital, Birmingham B16 8ET, UK.*

Regionalised variable theory (RVT) was developed to analyse spatially distributed properties that vary in a random, unpredictable manner. Early applications of its methods were in the mining industry to estimate the amount of metal/mineral in ore deposit. The central tool of RVT is the variogram which defines the pattern and spatial scale of the variation. Once the variogram has been modelled we can use the model parameters for estimation by kriging. Kriging is an optimal estimation procedure that uses the form of the spatial variation contained in the variogram to provide the best possible estimates with known confidence. The spatial variation can then be mapped isarithmically.

We are investigating the possibility of applying kriging to the incidence of childhood cancer. The problems we face relate to the low rates, the statistical distribution and the lack of continuity in the variation. This last problem has been faced by workers in diamond mining, yet they have found the methods of value for predicting high concentrations. We can use the more advanced technique of disjunctive kriging to transform the distribution using hermite phynomials. If the spatial variation of childhood cancer is second order stationary, the technique may be used further to set a critical threshold of incidence and to determine the probability of exceeding this value.

This technique is being applied to the data of the West Midlands Regional Children's Tumour Registry. This specialised registry contains data on all cases of childhood cancer in the West Midlands Health Authority Region. Cases diagnosed between 1975 an 1984 have been mapped and their distribution assessed using Poisson probability mapping. The results of this form of analysis are being compared with the assessment of clustering through the methods of RVT. A comparison of the results by both methods is given.

### Adult T-cell leukaemia/lymphoma (ATLL) in Brazil. Evidence for a new endemic area for HTLV-1

E. Matutes, M.P. de Olivera, T.S. Shulz, M.J. Andrada-Serpa, M.L. Calabro, R. Weiss & D. Catovsky

*Institute of Cancer Research, London, and Instituto Nacional de Cancer, Rio de Janeiro, Brazil.*

We describe a series of 12 patients with ATLL diagnosed over a period of 2 years in Rio de Janeiro. The disease had typical clinical features and malignant cells with a CD4 +, CD25 +, CD8- and CD7- membrane phenotype. This series includes an 18-month-old black infant whose mother and grandmother were HTLV-1 seropositive. Seven of the 12 patients were black or mulatto race and five were whites, four of Portuguese descent. Serological assays for HTLV-1 were positive in seven out of eight cases tested and these results were confirmed by Southern blotting of patients DNA using a full length HTLV-1 probe. The disease in Brazil is indistinguishible from ATLL in the other endemic areas, southwestern Japan and the Caribbean basin. There is independent seroepidemiological evidence for HTLV-1 infection in Brazil (Cortes *et al.*, *NEJM*, **320**, 1989, 953; Andrada-Serpa, *Int. J. Cancer*, 1990, in the press). This report confirms the presence of the T-cell malignancy caused by HTLV-1 in the Brazilian population. Studies on the mode of transmission of the retrovirus in Brazil and other South American countries are necessary.

### Identification of immunologically reactive epitopes on the GAG proteins of HTLV-1

S. Gledhill, S. Crae, R.S. Tedder, M. Robert-Guroff, D.E. Onions & R. Jarrett

*LRF Virus Centre, Glasgow University Veterinary School, Glasgow G61 1QH; Dept of Medical Microbiology, University College and Middlesex School of Medicine, London W1P 7PN; Laboratory of Tumour Cell Biology, NCI, NIH, Bethesda, MD 20892, USA.*

Using Western blot assays, cross-reacting antibodies to HTLV-1 gag proteins have been detected in sera from patients with cutaneous T-cell lymphoma (Ranki & Krohn, 1987, *Viruses in Human Cancer*, p.47) and from healthy blood donors (Schupbach *et al.*, 1983, *J. Exp. Med.*, **157**, 907). In addition some monoclonal antibodies raised against HTLV-1 p19 cross-react with normal and malignant tissues (Haynes *et al.*, 1983, *J. Exp. Med.*, **157**, 907).

In order to identify the immunologically reactive epitopes on the gag proteins of HTLV-1 we have synthesised overlapping peptides covering p19 and p24. We have identified an epitope near the carboxyl terminus of p19 recognised by a monoclonal antibody reactive with p19 but which also cross-reacts with other tissues (Robert-Guroff *et al.*, 1981, *J. Exp. Med.*, **154,** 1957). Sera from HTLV-1 seropositive patients show reactivity with two regions near the carboxyl terminus of p19.

We are investigating the epitopes recognised by HTLV-2 positive sera and those recognised by sera classified as HTLV-1 negative according to accepted criteria, which contain antibodies reactive with viral gag proteins. The results of these investigations should assist in determining the significance of cross-reactive antibodies in the sera of non HTLV-1-infected persons.

### Activation of HTLV-1 and HSV-1 promoter constructs by HHV-6

M. Campbell, S. McCorkindale & R. Everett

*Leukaemia Research Fund Virus Centre, Veterinary School, Glasgow G61 1QH; MRC Institute of Virology, Church Street, Glasgow G11 5JR, UK.*

Human herpes virus type 6 (HHV-6) is able to replicate in T cells and therefore may act as co-factor in the activation of Human T Cell Leukaemia Virus (HTLV-1). In order to study the interaction between the two viruses J Jhan cells were transfected with HTLV-1 LTR CAT constructs and infected with HHV-6. A 200-fold increase in CAT expression was observed compared to non infected controls. Time course studies showed activation of the HTLV-1 CAT construct to be due to HHV-6 proteins produced early in infection. Activation by HHV-6 and HTLV-1 *tax* is synergistic. In addition, an HTLV-1 CAT construct in which the 21bp repeat *tax* responsive sequences were deleted was still strongly induced by HHV-6 although basal levels of expression were greatly reduced showing HHV-6 and HTLV-1 *tax* to operate by different mechanisms. In order to determine if any basal regulatory elements are required for activation by HHV-6 a series of HSV-1 gD promoter deletion mutants are being studied.

### The seroepidemiology of HHV-6 in a case controlled study of leukaemia and lymphoma

D. Clark, F. Alexander, P. McKinney, R. Jarrett, R. Cartwright & D. Onions

*LRF Virus Centre, Dept Veterinary Pathology, University of Glasgow, Glasgow G61 1QH; LRF Clinical Epidemiology Centre, University of Leeds, Leeds LS2 9NG, UK.*

The association of human herpesvirus-6 (HHV-6) seropositivity and antibody titre with haematological malignancies was examined utilising 940 sera from a case controlled epidemiological survey.

In an indirect immunofluorescence assay 55% of the control sera were positive for HHV-6 antibody at a titre $\geqslant$1:40 and there was a significant increase in geometric mean titre (GMT) in females. Analysis of individual disease subtypes showed there was a strong association between HHV-6 seropositivity and titre in acute myeloid leukaemia, Hodgkin's disease (HD) and low-grade non-Hodgkin's lymphoma; the GMT ratios of cases to controls being 3.97, 2.94 and 5.2 respectively (95% CI).

These results may reflect altered patterns of HHV-6 replication or reactivation in these conditions. In HD, however, there was evidence for increased titres and seropositivity in young cases that lacked siblings in the family. It is possible that late infection by HHV-6 is occurring in these cases and the immune response to HHV-6 may play a role in the pathogenesis of HD.

### Virus involvement in Hodgkin's disease

S. Gledhill, A. Gallagher, E. Klee, D. Jones, A.S. Krajewski, D.E. Onions & R.F. Jarrett

*LRF Virus Centre, Veterinary School, Glasgow University, Glasgow; Southampton General Hospital, Southampton SO9 4XY; Department of Pathology, University of Edinburgh Medical School, Edinburgh, UK.*

Epidemiological studies have suggested that Hodgkin's Disease (HD) might have a viral aetiology. Herpes viruses have been implicated by serological studies showing elevated serum antibodies to EBV (Evans & Gutensohn, 1984, *Int. J. Cancer*, **34,** 149) and HHV-6 (Clark *et al.*, submitted) in HD cases. In addition, one report has described the detection of HTLV-1 by PCR in a case of HD (Duggan *et al.*, 1988, *Blood*, **71,** 1027).

In a series of 37 cases of HD which were analysed for the presence of immunoglobulin heavy chain (IgH) and T-cell receptor (TCR) gene rearrangements, we used Southern blot analysis to look for the presence of EBV and HHV-6 DNA. PCR was used to look for the presence of HTLV-1 DNA sequences.

Approximately 1/4 of the cases showed IgH gene rearrangements, TCR genes were germline. We did not detect HHV-6 DNA in any of the samples. Of 18 samples analysed for the presence of EBV DNA 6 were positive. Detection of EBV did not correlate with the detection of IgH gene rearrangements. HTLV-1 sequences were not amplified in any of 20 samples analysed using PCR.

These results suggest that EBV may play a role in the pathogenesis of some cases of HD.

### P-glycoprotein (PGp) expression and glutathione (GSH) content in human leukaemia cells determined by flow-cytometry (FCM)

M.R. Müller, P.R. Twentyman, E. Lambert, H. Cox, J.V. Watson, T.P. Baglin & J.H.K. Rees

*MRC Clinical Oncology and Radiotherapeutics Unit and University Department of Haematology, Cambridge, UK.*

PGp expression and GSH content can be determinants of cytotoxic drug resistance in cancer cells. We have established optimal conditions for measuring these parameters in human leukaemia cells by FCM. To determine PGp expression isolated cells were stained with the monoclonal antibody MRK16 and FITC-labelled rabbit-antimouse serum and fixed in paraformaldehyde. Cells were also stained with propidium iodide and a DNA-histogram simultanously recorded. To date, 20 patients with haematological malignancies have been investigated. Three patients with clinically resistant AML, CLL and CML in blast crisis had more than 40% positive cells. Four non-responsive and 14 untreated patients were negative for PGp. There was no direct relationship between the *in vitro* responsiveness to adriamycin determined by the MTT assay and PGp content in patients studied to date. We have previously observed a good correlation between the cellular GSH content of H69 human lung cancer cells after BSO-depletion as determined by FCM using the probe monochlorobimane and by spectrophotometry (Tietze-assay). This correlation was also found in determination of GSH-content in isolated lymphocytes from 14 patients with CLL. Two-fold higher levels of cellular GSH were seen in 4

patients with clinically resistant CLL (88 ng per $10^5$ cells, 736 fluorescence units (FU)) compared to six untreated CLL patients (37.7 ng per $10^5$ cells, 443 FU) and eight healthy individuals (46.5 ng per $10^5$ cells, 482 FU). Interestingly, these non-responsive patients with elevated cellular GSH content were negative for PGp-staining. Studies are underway to investigate the role and relationship of PGp and GSH to cytotoxic drug sensitivity determined in the MTT-assay and to clinical responsiveness.

### Multidrug resistance in chronic lymphatic leukaemia

P. Cumber, R.A. Padua, J. Holmes, G. Carter & A. Jacobs

*LRF Preleukaemia Unit, University of Wales College of Medicine, Heath Park, Cardiff, UK.*

Chronic lymphocytic leukaemia (CLL) is generally a disease of slow progression and is resistant to cytotoxic treatment. Drug resistance mechanisms may therefore be potentially relevant in CLL. The multidrug resistance gene *mdr* 1 encodes a transmembranous p-glycoprotein which effectively acts as an efflux pump leading to a decreased intracellular concentration of cytotoxic drugs. We have studied 34 patients with CLL (seven untreated, 27 treated); DNA was screened for gene amplification, RNA for increased expression of *mdr* 1 and p-glycoprotein for expression using FACS analysis and a monoclonal antibody to the external epitope (MRK-16). Normal control lymphocytes from 10 healthy volunteers were prepared and levels of RNA expression and protein in these samples were found to be low relative to vinblastine sensitive and resistant cell lines. Eighteen patients (four untreated, 14 treated) were observed to have *mdr* 1 RNA expression levels above the normal range. No DNA amplification was found in any samples investigated. The p-glycoprotein levels were elevated in five (one untreated and four treated) of these CLL patients. The discrepancy between the high incidence of increased RNA levels and apparently low protein expression in CLLs may be explained by masking of p-glycoprotein on the cell surface membrane.

### The identification of drug resistance and cross-resistance patterns in acute myeloid leukaemia (AML)

J.M. Sargent, C.G Taylor, A.W. Elgie & J.K. Wilson

*Dept of Haematology, Pembury Hospital, Pembury, Kent TN2 4QJ, UK.*

The identification of drug resistance in individual patients before treatment would be a therapeutic advance in the management of AML. The advantages of short-term *in vitro* drug sensitivity assays are now being recognised. We have validated the use of the rapid and simple MTT assays for chemosensitivity testing of blast cells from AML (Sargent & Taylor, 1989, *Br. J. Cancer*, **60**, 206). There is a good reproducible variation in drug effect between patients. The assay results correlated with the clinical response in 21 of 23 cases. In 16 patients with *de novo* disease tested against up to seven drugs, one showed resistance to all agents tested, four were resistant to doxorubicin, one to mitoxantrone and eight to cytosine arabinoside. Of the four resistant to doxorubicin, three were sensitive to mitoxantrone. Four patients were tested against doxorubicin, mitoxantrone and daunorubicin. The one sample resistant to doxorubicin in this group was also resistant to mitoxantrone but sensitive to daunorubicin. This assay can not only predict *de novo* resistances but, because of its simplicity and efficiency, it can be used throughout the course of the disease to detect changes in sensitivity. Sequential analyses in two patients over several months demonstrated increasing resistance to doxorubicin. The test is a suitable method to study the *in vitro* effect of potential resistance modifiers, for example the calcium antagonist verapamil significantly increased sensitivity to doxorubicin. Few cells are required for the MTT assay so, in addition to dose response curves for single agents it was possible in 11 patients, to construct a model to mimic the standard combination drug regimes. No evidence of synergism was found. To conclude, the reliability, speed and simplicity of the MTT assay facilitates the study of drug resistance and resistance modifiers in AML.

### Alternative anthracyclines and resistance modifiers in human leukaemias. An *in vitro* study using the MTT assay

P.R. Twentyman, M. Müller, E. Lambert & J.H.K. Rees

*MRC Clinical Oncology and Radiotherapeutics Unit and University Department of Haematology, Hills Road, Cambridge CB2 2QH, UK.*

In studies using multidrug resistant (MDR) cell lines, we have developed two strategies for overcoming resistance. The first involves the identification of alternative drugs which retain activity in MDR cells and the second uses resistance modifiers (RMs) to restore sensitivity. These approaches have now been tested in human leukaemias using peripheral blood cells and *in vitro* chemosensitivity testing using a 4 day MTT assay (Twentyman *et al.*, *Br. J. Haematol.*, 1989,.**71**, 19). We have determined sensitivity to adriamycin (ADM), vincristine (VCR) and melphalan (MEL), the alternative anthracyclines aclacinomycin A (ACL) and the Japanese morpholinyl compound MX2, together with resistance modifiers verapamil (VRP, 2 μM) and cyclosporin A (CYA, 0.8 μM). To date 10 samples of normal lymphocytes (NL), 26 CLL, 3CML and 2AML have been studied. Sensitisation ratios (i.e. ratio of cytotoxic drug ID50s in absence/presence of RM) obtained are shown below in the table.

| | *ADM* | | *VCR* | | *MEL* | | *ACL* | | *MX2* | |
|---|---|---|---|---|---|---|---|---|---|---|
| | *VRP* | *CYA* | *VRP* | *CYA* | *VRP* | *CYA* | *VRP* | *CYA* | *VRP* | *CYA* |
| NL | 1.1 | – | 1.1 | – | – | – | – | – | – | – |
| CLL | 1.4 | 1.2 | 2.6 | 3.1 | 1.0 | – | 1.0 | 1.9 | 1.1 | 1.1 |
| AML + CML | 1.5 | 1.7 | 1.6 | 1.9 | 0.9 | – | 1.0 | 0.9 | 1.1 | 0.9 |

The relative efficacies of ACL and MX2 (compared to ADM) were higher for AML + CML samples than for CLL or NL. These data indicate that RMs potentiate the potency of 'classic' MDR-type drugs (ADM,VCR) in leukaemic cells but not in NL. No such potentiation is seen for MEL or the 'low-resistance' drugs ACL and MX2. Such studies may facilitate the optimisation of chemotherapy for leukaemias on an individual patient basis.

### Use of the MTT assay to assess drug resistance patterns in acute myeloid leukaemia (AML)

J.K. Phillips

*Liverpool University Department of Haematology, Royal Liverpool Hospital, Prescot Street, Liverpool L69 3BX, UK.*

Cytotoxics affected by multidrug resistance (MDR) *in vitro* are major components of AML chemotherapy, but the relevance of this phenomenon to clinical resistance is unclear. Using the MTT assay this study aims to establish in AML: (1) the range of dose–response curves to four MDR (mitozantrone, adriamycin, VP16, vincristine) and two non-MDR cytotoxics (cytosine, thioguanine); (2) to what extent *in vitro* sensitivity correlates with clinical response; and (3) the frequency of the MDR phenotype. Blasts purified from peripheral blood/bone marrow in 23 patients showed wide ranges of ID50s (cytotoxic concentrations giving 50% reduction in optical density (OD) after 72 h cytotoxic exposure for all six drugs. No significant difference was seen between relapsed ($n = 7$) and untreated ($n = 16$) groups. Percentage reduction in OD at cytotoxic concentrations corresponding to approximately 1/10 peak achievable plasma levels (>70% = sensitive, ⩽70% = resistant) Bird *et al.*, *Cancer*, 1988, **61**, 1104) correctly predicted clinical response in only four of 12 samples for which relevant *in vivo* and *in vitro* data are available. In 12 samples all six drugs were tested in the presence and absence of 6.6 μM verapamil (VER). Four of these (two relapsed, two untreated) showed a clear increase in sensitivity to MDR drugs in the presence of VER but in none were all four drugs affected. *In vitro* sensitivity to non-MDR drugs was not increased by VER.

*In vitro* sensitivity to component drugs measured by MTT assay predicts poorly for clinical response to combination chemotherapy. VER modification of MDR drug sensitivity *in vitro* is seen in both untreated and relapsed AML.